# Perception **of treatments with self-ligating and conventional brackets in peruvian orthodontists**

**DOI:** 10.21142/2523-2754-1203-2024-206

**Published:** 2024-09-17

**Authors:** Diego Alonso Huayta-Garcia, Kilder Maynor Carranza-Samanez, Julissa Amparo Dulanto-Vargas

**Affiliations:** 1 School of Dentistry, Universidad Científica del Sur. Lima, Perú. 100030107@cientifica.edu.pe Universidad Científica del Sur School of Dentistry Universidad Científica del Sur Lima Peru 100030107@cientifica.edu.pe; 2 Research Group in Dental Sciences, School of Dentistry, Universidad Científica del Sur. Lima, Perú. kcarranza@cientifica.edu.pe, jdulanto@cientifica.edu.pe Universidad Científica del Sur Research Group in Dental Sciences School of Dentistry Universidad Científica del Sur Lima Peru kcarranza@cientifica.edu.pe jdulanto@cientifica.edu.pe

**Keywords:** perception, orthodontic brackets, orthodontists, percepción, soportes ortodónticos, ortodoncistas

## Abstract

**Introduction:**

Orthodontists' perception of bracket techniques plays a significant role in planning, allowing critical evaluation of the patient's facial aesthetics.

**Objective:**

To compare the perception of Peruvian orthodontists regarding treatments with self-ligating and conventional brackets.

**Methods:**

A questionnaire was applied to 168 orthodontic specialists (53% men, average professional experience 9 years) to evaluate preferences for treatment phases, benefits of patient consultation according to the type of bracket, experience with self-ligating brackets, and demographic and clinical characteristics (sex, years of experience, volume of care and length of experience). The Kruskal-Wallis Test and Chi-square test were used with P < 0.05.

**Results:**

The total preference for self-ligating brackets (48.9%) was higher than conventional brackets (18.8%). Self-ligating brackets were preferred in most treatment phases (46.4%-63.7%) but not in completion and finishing, in which conventional brackets were preferred. Most orthodontists preferred self-ligating brackets (40.5%-60.7%) over conventional brackets (4.2%-14.3%) due to patient comfort, oral hygiene, and total treatment and appointment time control, but not the cost. The orthodontists reported SLB having mastered their technique in <10 cases (59.5%), the experience of <2 years (45.2%), applying control times of 4-9 weeks (78.6%), and feeling comfortable with their use (89.3%). Preferences were not associated with sex (*P* > 0.05) but rather with years of professional experience (*P* < 0.05).

**Conclusions:**

Peruvian orthodontists preferred self-ligating brackets over conventional brackets in most treatment phases associated with user comfort and oral hygiene management and treatment/control time, and professional experience. However, some factors, such as cost-effectiveness, counteracted this preference.

## RESUMEN

**Introducción:**

La percepción de los ortodoncistas sobre las técnicas de *brackets* cumple un papel importante en la planificación, al permitir una evaluación crítica de la estética facial del paciente.

**Objetivo:**

Comparar la percepción de tratamientos con *brackets* autoligantes y convencionales en ortodoncistas peruanos.

**Métodos:**

Un cuestionario fue aplicado a 168 especialistas en ortodoncia (53% varones, experiencia profesional promedio 9 años) para evaluar las preferencias por fases de tratamiento, beneficios de la consulta del paciente según el tipo de *bracket*, experiencia con *brackets* autoligantes, características demográficas y clínicas (sexo, años de experiencia, volumen de atención y tiempo de experiencia). Las pruebas de Kruskal-Wallis y chi-cuadrado se utilizaron con un p < 0,05.

**Resultados:**

La preferencia total de los *brackets* autoligantes (48,9%) fue mayor frente a los *brackets* convencionales (18,8%). Los *brackets* autoligantes fueron preferidos en la mayoría de las fases de tratamiento (46,4%-63,7%), pero no en la finalización y acabado, en las que se prefirieron los convencionales. La mayoría de los ortodoncistas prefirieron los *brackets* autoligantes (40,5%-60,7%) a los convencionales (4,2%-14,3%) debido a la comodidad e higiene oral del paciente, y el tiempo total de tratamiento y citas de control, pero no por el precio. Los ortodoncistas refirieron haber dominado la técnica de los *brackets* autoligantes en <10 casos (59,5%), experiencia < 2 años (45,2%), aplicando tiempos de control de 4-9 semanas (78,6%), y sentirse cómodo con su uso (89,3%). Las preferencias no se asociaron al sexo (p > 0,05), sino con los años de experiencia profesional (p < 0,05).

**Conclusiones:**

Los ortodoncistas peruanos prefirieron los *brackets* autoligantes sobre los *brackets* convencionales en la mayoría de las fases de tratamiento, debido a factores asociados con la comodidad e higiene oral del usuario, el manejo y tiempo de tratamiento/control, y la experiencia profesional. Sin embargo, hubo otros factores que contrarrestaron la preferencia, como la rentabilidad en el costo.

## INTRODUCTION

Orthodontic treatment with conventional brackets (CB) is characterized by the contact between brackets and the metallic archwire using ligatures and elastic modules. This modality has been the most commonly used for several decades; however, it presents disadvantages such as the inability to provide complete arch-to-bracket engagement and relatively high friction. This is proportional to the inflammatory response of periodontal tissues and dental roots ^1^^,^^2^.

New orthodontic techniques, such as self-ligating brackets (SLB), have resulted in reduced biological response. This technique diminishes friction and achieves complete arch engagement, facilitating alignment, leveling, and space closure in less time ^3^^,^^4^. However, despite this, the reduction in friction is not evident when compared to CB in severe malocclusions and during the final stages of treatment ^5^^,^^6^.

Orthodontists have various possibilities for choosing orthodontic techniques and materials. This enables them to optimize therapies, gain experience, and offer alternatives to patients ^7^. Professional perception of bracket techniques plays a significant role in planning, allowing critical evaluation of the patient's facial aesthetics from the orthodontist's perspective ^8^^,^^9^.

The clinical perception of orthodontic professionals regarding bracket systems has previously been studied, showing that CB was preferred due to the cementing technique, the final outcome, and cost-effectiveness. Meanwhile, SLB was chosen because it was considered to improve patient hygiene and comfort, reduce chair time, decrease friction, speed up alignment, and shorten treatment duration ^10^^-^^14^.

From the literature review conducted in this study, no information was found about the opinions of Peruvian orthodontists regarding the use of brackets ranging from conventional to more advanced technology. Therefore, it is essential to determine the current state of using these bracket systems in Peru. Hence, this study aimed to compare the perception of orthodontic treatments using self-ligating brackets and conventional brackets among a sample of orthodontists in Peru.

## MATERIALS AND METHODS

### Sample population

This cross-sectional study was approved by the Institutional Research Ethics Committee of the Universidad Científica del Sur (N°. 334-CIEI-CIENTÍFICA-2021) and performed following the STROBE guidelines and the Declaration of Helsinki. Informed consent was obtained from each participant after explaining the objective of the study. The study population consisted of 963 orthodontic specialists registered with the Colegio Odontológico del Perú. The sample comprised 168 Peruvian orthodontists (PO) who completed a questionnaire in 2021 (response rate = 17.4%). The inclusion criterion was to have the cognitive capacity to answer an individual survey and being members of the “Asociación Iberoamericana de Ortodoncistas” (AIO) del Perú.

### Perceptions questionnaire

A structured self-reporting questionnaire consisting of 20 questions was used to assess the perception and experience of PO regarding orthodontic treatment (13 items), as well as their demographic and clinical practice characteristics (7 items). The questionnaire was adapted from a previous study ^12^ through certified translation and was reviewed by five expert faculty members and orthodontic specialists. 

The perception variable was distributed across four dimensions: preferences for types of brackets according to treatment phases (five items), patient benefits preferences (four items), clinical benefits preferences (four items), and experience with SLB (four items). The perception questionnaire considered seven active questions and 13 passive questions. The response options for the first three dimensions were: a) NP - no preference, b) CB, and c) SLB, while the last dimension had multiple alternatives.

### Study variables

The characteristics of the OP included data on sex, years of professional experience, patient volume, length of experience with SLB, and appointment times for SLB and CB. Participants received an invitation to the virtual questionnaire conducted via Google Forms sent to the email addresses provided by the AIO-Perú. Only orthodontists who consented and completed the entire questionnaire were included.

### Statistical analysis

Descriptive analysis included frequencies (n), percentages (%), medians (Me), and interquartile ranges (IQR). Years of professional experience showed a non-normal distribution analyzed using the Kolmogorov-Smirnov test. Inferential statistics included the Chi-square test to compare perceptions by sex and the Kruskal-Wallis test to compare perceptions by years of professional experience. Data were analyzed using SPSS v.25.0 software at a significance level of 0.05.

## RESULTS

The sample of PO was characterized by a similar distribution of sex (53% males and 47% females), 9 years of professional experience (IQR = 6), ≤30% patient volume treated with SLB (74.4%), <2 years of experience with SLB (45.2%), and appointments every 4 to 5 weeks with CB (92.2%) and 4-9 weeks with SLB (78.6%) (Table 1).

**Table 1:** Characteristics of the sample of Peruvian orthodontists.

Secondary variables	Parameters	n	%
Sex	Male	89	53%
	Female	79	47%
SLB care volume	0% to 30%	125	74.4%
	31% to 70%	37	22.0%
	71% to 100%	6	3.6%
Time of experience with SLB	< 2 years	76	45.2%
	2 to 10 years	47	28.0%
	> 10 years	45	26.8%
Time of experience with SLB	Median (IQR)	9	(6)
SLB experience time	< 2 years	47	28%
	2 to 10 years	76	45.2%
	> 10 years	45	26.8%
Control times for CB	4 to 5 weeks	155	92.2%
	6 to 7 weeks	6	3.6%
	8 to 9 weeks	5	3.0%
	≥10 weeks	2	1.2%
Control times for SLB	4 to 5 weeks	42	25.0%
	6 to 7 weeks	33	19.6%
	8 to 9 weeks	57	34.0%
	≥10 weeks	36	21.4%

SLB, self-ligating brackets. CB, conventional brackets.

The length of professional experience showed a positive association with patient volume (≥71%) and shorter appointment intervals with SLB (≤5 weeks) (*P* = 0.027 and *P* = 0.001, respectively), but not with the length of experience with SLB (p = 0.133) or CB (*P* ≥ 0.321). Sex did not correlate with the characteristics of treatment using SLB or CB (*P* ≥ 0.05) (Table 2).

**Table 2:** Characteristics of orthodontic treatments with brackets according to sex and years of professional experience in Peruvian orthodontists.

Secondary variables	Values	Years of experience			Sex, n (%)		
		Me	IQR	P value†	Me	IQR	P value‡
SLB care volumen	0% to 30%	10	7	0.027*	63 (70.8%)	62 (78.48%)	0.251
	31% to 70%	7	2.5		21 (23.6%)	16 (20.25%)	
	71% to 100%	9.5	5		5 (5.6%)	1 (1.27%)	
Time of experience with SLB	< 2 years	11	5	0.133	24 (0.27%)	23 (29.1%)	0.466
	2 to 10 years	9	6		44 (49.4%)	32 (40.5%)	
	> 10 years	9	5.5		21 (23.6%)	24 (30.4%)	
Control times for CB	4 to 5 weeks	9	6	0.321	84 (94.4%)	71 (89.9%)	0.439
	6 to 7 weeks	10	7		3 (3.4%)	3 (3.8%)	
	8 to 9 weeks	7	3.5		2 (2.2%)	3 (3.8%)	
	≥10 weeks	10	4		0 (21.3%)	2 (21.5%)	
Control times for SLB	4 to 5 weeks	12	6	0.001*	23 (25.8%)	19 (24.1%)	0.319
	6 to 7 weeks	9	5.5		13 (14.6%)	20 (25.3%)	
	8 to 9 weeks	7	4		34 (38.2%)	23 (29.1%)	
	≥10 weeks	9.5	6		19 (21.3%)	17 (21.5%)	

SLB, self-ligating brackets. CB, conventional brackets. Me: Median. IQR: Interquartile range. †Kruskal-Wallis test. ‡Chi-square test. *P < 0.05

The overall preference and patient/clinic benefits preference among PO for SLB (44% to 48.9%) were statistically higher compared to CB (9.4% to 26.2%) (*P* < 0.001). SLB were also preferred in most treatment phases (46.4% to 63.7%; P < 0.05) except for finishing and detailing, in which it was similar to CB (*P* = 0.143). SLB (40.5% to 60.7%) were preferred over CB (4.2% to 14.3%) for patient comfort and hygiene, appointment times, and total treatment duration (*P* < 0.001), but not in terms of price in which CB was preferred (P < 0.001). The greater preference for non-extraction dental crowding, long-term stability, fewer emergency appointments, and better dental assistant work showed a trend towards both systems (42.3% to 53.6%) or SLB (33.9% to 49.4%) compared to CB (4.2% to 14.3%) (*P* < 0.001) (Table 3).

**Table 3:** Perception regarding preferences of orthodontic treatment with brackets among Peruvian orthodontists.

Dimensional preferences	Brackets, n (%)		SLB	P value
	CB	NP		
Total preference	411 (18.8%)	704 (32.2%)	1069 (48.9%)	<0.000*
Preferences for treatment phases	172 (20.5%)	249 (29.6%)	419 (49.9%)	<0.000*
Start	22 (13.1%)	39 (23.2%)	107 (63.7%)	<0.000*
Alignment	38 (22.6%)	43 (25.6%)	87 (51.8%)	<0.000*
Space closure	43 (25.6%)	47 (28%)	78 (46.4%)	<0.000*
Completion and finishing	51 (30.4%)	49 (29.2%)	68 (40.5%)	0.143
Patient benefit preferences	63 (9.4%)	255(37.9)	354(52.7%)	<0.000*
Comfort	16 (9.5%)	51 (30.4%)	101 (60.1%)	<0.000*
Oral hygiene	16 (9.5%)	50 (29.8%)	102 (60.7%)	<0.000*
Tooth crowding	7 (4.2%)	78 (46.4%)	83 (49.4%)	<0.000*
Long term stability	24 (14.3%)	76 (45.2%)	68 (40.5%)	<0.000*
Preferences for benefits of the consultation	176 (26.2%)	200(29.8)	296(44%)	<0.000*
Control time	64 (38.1%)	29 (17.3%)	75 (44.6%)	<0.000*
Treatment time	15 (8.9%)	54 (32.1%)	99 (58.9%)	<0.000*
Emergency appointment	18 (10.7%)	71 (42.3%)	79 (47%)	<0.000*
Dental assistant job	21 (12.5%)	90 (53.6%)	57 (33.9%)	<0.000*
Price	76 (45.2%)	27 (16.1%)	65 (38.7%)	<0.000*

SLB, self-ligating brackets. CB, conventional brackets. NP, no preference. One sample Chi-square test. *P < 0.05

The majority of PO required <10 cases to master the SLB technique (59.5%) and felt comfortable with its use (89.3%) (*P* < 0.001). A sample of 24.4% of orthodontists who went through the experience of suspending SLB considered that it did not show advantage to justify the high cost (Table 4).

**Table 4:** Perception regarding experiences of orthodontic treatment with SLB among Peruvian orthodontists.

SLB experiences	Items	n	%	P value
Orthodontist domain	< 10 cases	100	59.5%	<0.000*
	From 10 to 30 cases	43	25.6%	
	> 30 cases	7	4.2%	
Orthodontist comfort	No	18	10.7%	<0.000*
	Yes	150	89.3%	
Marketing strategy	No	72	42.9%	0.064
	Yes	96	57.1%	
SLB suspension	Better results not obtained compared to CB	22	13.1%	<0.000*
	SLB showed no advantage to justify the high cost	41	24.4%	
	I do not like SLB	3	1.8%	
	Patients do not like SLB	4	2.4%	
	Other reasons	98	58.3%	

SLB, self-ligating brackets. CB, conventional brackets. One sample Chi-square test. *P < 0.05

The perception regarding preferences between orthodontic treatment types by phases and/or patient or clinical benefits did not correlate with the length of professional experience or the sex of the PO (P ≥ 0.309) (Table 5).

**Table 5:** Comparison of perception regarding preferences for orthodontic treatment with brackets according to sex and years of experience among Peruvian orthodontists.

Preferences	Brackets	Years of experience			Sex, n (%)		P value‡
		Me	IQR	P valor†	Male	Female	
Preferences for treatment phases							
Start	CB	10.5	6	0.194	12 (7.1%)	10 (6.0%)	0.855
	SLB	9	6		55 (32.7%)	52 (31.0%)	
	NP	9	6		22 (13.1%)	17 (10.1%)	
Alignment	CB	11	4.5	0.301	20 (11.9%)	18 (10.7%)	0.792
	SLB	9	6		48 (28.6%)	39 (23.2%)	
	NP	9	6		21 (12.5%)	22 (13.1%)	
Space closure	CB	11	4	0.076	20 (11.9%)	23 (13.7%)	0.340
	SLB	9	6		46 (27.4%)	32 (19.0%)	
	NP	8	6		23 (13.7%)	24 (14.3%)	
Completion and finishing	CB	10	5	0.113	23 (13.7%)	28 (16.7%)	0.880
	SLB	9.5	6.75		43 (25.6%)	25 (14.9%)	
	NP	8	5		23 (13.7%)	26 (15.5%)	
Patient benefit preferences							
Comfort	CB	11	5.5	0.151	8 (4.8%)	8 (4.8%)	0.893
	SLB	9	6		55 (32.7%)	46 (27.4%)	
	NP	9	6		26 (15.5%)	25 (14.9%)	
Oral hygiene	CB	12	6	0.140	9 (5.4%)	7 (4.2%)	0.427
	SLB	9	5.25		50 (29.8%)	52 (31.0%)	
	NP	9	6		30 (17.9%)	20 (11.9%)	
Tooth crowding	CB	9	5	0.978	3 (1.8%)	4 (2.4%)	0.604
	SLB	9	6		47 (28.0%)	36 (21.4%)	
	NP	9	6		39 (23.2%)	39 (23.2%)	
Long term stability	CB	10.5	4	0.378	11 (6.5%)	13 (7.7%)	0.622
	SLB	9	6		35 (20.8%)	33 (19.6%)	
	NP	9	6		43 (25.6%)	33 (19.6%)	
Preferences for benefits of the consultation							
Control time	CB	9	6	0.095	34 (20.2%)	30 (17.9%)	0.842
	SLB	8	5		41 (24.4%)	34 (20.2%)	
	NP	11	7.5		14 (8.3%)	15 (8.9%)	
Treatment time	CB	10	6	0.569	9 (5.4%)	6 (3.6%)	0.710
	SLB	9	6		50 (29.8%)	49 (29.2%)	
	NP	10	8		30 (17.9%)	24 (14.3%)	
Emergency appointment	CB	11	4.5	0.336	8 (4.8%)	10 (6.0%)	0.363
	SLB	9	6		39 (23.2%)	40 (23.8%)	
	NP	8	6		42 (25.0%)	29 (17.3%)	
Dental assistant job	CB	10	6	0.103	9 (5.4%)	12 (7.1%)	0.575
	SLB	11	7		30 (17.9%)	27 (16.1%)	
	NP	8	6		50 (29.8%)	40 (23.8%)	
Price	CB	9	6	0.542	38 (22.6%)	38 (22.6%)	0.309
	SLB	9	6		39 (23.2%)	26 (15.5%)	
	NP	10	7		12 (7.1%)	15 (8.9%)	

SLB, self-ligating. CB, conventional brackets. NP, no preference. Me: Median. IQR: Interquartile range. †Kruskal-Wallis test. ‡Chi-square test. *P < 0.05

## DISCUSSION

The field of orthodontics has evolved due to the development of new techniques aimed at greater effectiveness in meeting the functional and aesthetic needs of the population ^7^. This present study compared the perception of treatments with SLB and CB among a sample of PO. The primary result showed differences in total preferences for bracket types, in which almost half leaned towards SLB and only a fifth towards CB, while the rest had no preference.

Previous comparative studies that used questionnaires to assess preferences for bracket types also found a preference for using SLB compared to CB among orthodontists in populations from Saudi Arabia ^10^, Brazil ^11^, the United States ^12^, India ^13^, and Romania ^14^. Similar to this research, these results varied according to the clinical situation, the cost/benefit factor for the patient, or the profitability of the treatment.

In the secondary results of this study, it was found that SLB was preferred over CB by the majority of PO in most phases of treatment concerning patient-related benefits in comfort and hygiene and clinical benefits due to treatment time and control appointments. PO reported that SLBs were used in up to 30% of their treatments, control appointments were conducted every four to nine weeks, and they mastered the technique in less than ten cases and felt comfortable using it, but it was not the main objective of their clinical marketing strategy. More professional experience was associated with higher patient volumes and shorter control appointment intervals with SLB.

The preferences for SLB in this study coincided with other populations in the initial ^11^^-^^13^, alignment ^10^^-^^13^, and space closure phases ^10^^,^^12^^,^^13^. This result aligns with the biomechanics of the system that influences the efficiency and speed of action of this type of bracket ^5^^,^^15^, although some studies reported that there was no significant superiority of SLB over CB in alignment ^16^ or space closure ^16^^,^^17^. The preference in the finishing and detailing phase in this study was similar with both systems, as mentioned in a systematic review ^16^, but differed from other populations that leaned towards SLB ^10^^,^^13^ and CB ^11^^,^^12^.

The present results of preferences for SLB coincided with several studies regarding patient benefits, such as less discomfort or better oral hygiene ^10^^,^^12^^,^^14^, although the preference was similar for both systems in cases of avoiding extraction due to crowding or long-term relapse ^10^^,^^12^. Other studies also coincide with our result of clinical benefits for SLB related to appointment times ^10^^,^^12^ and total treatment duration ^13^, CB for providing better profitability ^10^^,^^12^^-^^14^, and no preference regarding fewer emergency appointments ^10^^,^^12^ and improved dental assistant work ^10^. However, other studies described no preference regarding treatment duration ^10^^,^^12^^,^^13^ or dental assistant work ^12^.

Previous systematic reviews mention SLB associated with a lower dental plaque index and halitosis ^18^^,^^19^, but the evidence was inconclusive in differentiating microbial colonization between SLB and CB ^20^. It is relevant to consider that complexity in cases of crowding might be a challenge for sliding mechanics during space closure, creating friction and pain during treatment ^5^. According to previous reviews, SLB or low-friction attachments were associated with less pain and a lesser impact on patients' quality of life in oral health ^2^^,^^5^. Regarding time, although some studies reported no difference between the types of brackets studied ^5^^,^^6^^,^^9^^,^^16^, this might contradict the presentations mentioned by distributors regarding the advantages of SLB products ^5^^,^^16^. Nevertheless, influential factors might limit this time efficiency, such as severe crowding treatments, extraction cases, patient cooperation, and the complexity of malocclusion ^4^^,^^6^^,^^9^.

The POs in this study were supported by different populations of orthodontists who reported up to 30% of their treatments using SLB ^10^^-^^12^, less than two years of experience with SLB ^10^, control appointments every four to five weeks with CB ^10^ and four to nine weeks with SLB ^10^^,^^12^ mastering the technique in less than ten cases ^10^^,^^11^, feeling comfortable using SLB. Still, these were not the primary reasons for the marketing strategy of their clinics ^10^. On the other hand, more professional experience in the PO of this study might have favored a higher number of SLB treatments. However, they tended to apply the exact control appointment times for CB and SLB and did not have preferences for one bracket system over another.

In comparison with the present study, the study on American orthodontists reported more experience with CB from two to ten years ^12^ and longer control appointment intervals of six to seven weeks with CB compared to SLB ^12^. This might be due to the historical approach of manufacturing the system. Our results in PO cannot be generalized due to the low response rate. However, it represents progress in studies of the opinions of orthodontists, a specialty that was the first to form in the field of Health Sciences and has 26 years of development in Peru.

### Limitations:

The survey was reviewed by specialist orthodontists, was formulated online, and was of adequate length to incentive response. However, the response rate was relatively low despite mass mailing to members of the orthodontic society. On the other hand, the multiple questions depended on the participants' memory, which could generate impressions of the information. It is possible that interest in the topic generated a self-selection bias in the participants, which could have influenced the result. For this reason, it is recommended that future studies involve larger samples and longitudinal designs to improve the precision of the data.

## CONCLUSIONS

Considering the limitations of this study, it can be concluded that Peruvian orthodontists preferred self-ligating brackets over conventional brackets in the initial treatment phases, alignment, and space closure. Preferences were associated with less patient discomfort and improved oral hygiene, better management and treatment time, and greater professional experience. However, factors countered their choice, such as higher costs and less professional experience.
